# The Staging Model in Psychosis and Preventive Interventions: An Emerging Field With Debates on Conceptual Models and Interpretation of the Evidence Base

**DOI:** 10.1111/eip.70185

**Published:** 2026-06-22

**Authors:** Mark van der Gaag, Cristina Mei, Barnaby Nelson, Maximus Berger, Marija Krcmar, G. Paul Amminger, Andreas Bechdolf, Paul Hutton, Pim Cuijpers, Alison R. Yung, Patrick D. McGorry

**Affiliations:** ^1^ Department of Clinical, Neuro and Developmental Psychology, Amsterdam Public Health Research Institute Vrije Universiteit Amsterdam Amsterdam the Netherlands; ^2^ Parnassia Psychiatric Institute The Hague the Netherlands; ^3^ Orygen Parkville Victoria Australia; ^4^ Centre for Youth Mental Health The University of Melbourne Parkville Victoria Australia; ^5^ Clinical Division of Social Psychiatry, Department of Psychiatry and Psychotherapy Medical University of Vienna Vienna Austria; ^6^ Comprehensive Center for Clinical Neurosciences and Mental Health Medical University of Vienna Vienna Austria; ^7^ Department of Psychiatry, Psychotherapy and Psychosomatic Medicine Incorporating FRITZ Am Urban and Soulspace Vivantes Hospital Am Urban and Im Friedrichshain Berlin Germany; ^8^ Department of Psychiatry and Psychotherapy, CCM Charité‐Universitätsmedizin Berlin Germany; ^9^ German Center of Mental Health (DZPG) Berlin Germany; ^10^ School of Health & Social Care Edinburgh Napier University Edinburgh Scotland; ^11^ School of Health Sciences University of Manchester Manchester UK; ^12^ Institute for Mental and Physical Health and Clinical Translation (IMPACT) School of Medicine, Deakin University Geelong Victoria Australia

**Keywords:** clinical high risk, cognitive behavioral therapy, prevention, psychosis, staging model

## Abstract

**Aims:**

The staging model in psychiatry is heuristic and evolving, provoking debate and stimulating alternative views. This paper reviews major controversies in the field of staging and prevention of psychosis.

**Methods:**

We review the literature on the classification model, the continuum model and the dynamic systems model applied to psychiatry. Other controversies are stigmatisation of early detection, the prevention paradox and the interpretation of data on prevention of transition to psychosis.

**Results:**

DSM classifications still lack validity. The continuum model supports preclinical stages but has no discrete conversions to different stages of illness. The emerging dynamic systems model builds on the interaction of symptoms eliciting networks of symptoms, while at the same time it explains sudden catastrophic shifts (transitions) between different states of equilibrium. Stigmatisation of early psychosis can be balanced against the benefits of early interventions. The prevention paradox states that only a minority of first episode cases arises from the high‐risk group. However, there are many reasons to combine indicated prevention with population‐based preventive interventions. A long‐lasting controversy is on the interpretation of the research data. Several meta‐analyses are reviewed on methodological issues.

**Conclusion:**

The staging model is adequate to conceptualise psychiatric disorders and the progress over time in more chronic stages. No one is suggesting that help‐seeking patients with an ARMS should be denied access to psychosocial treatment and it is recommended and necessary to continue building age and stage specific services to treat them and continue with research into risk factors and helpful interventions.

## 1 Introduction

The staging model of cancer in oncology has preclinical stages and progression to several clinical stages with different treatment approaches in different stages. It has been recognised since the time of Kraepelin that psychotic illnesses, notably schizophrenia, are also preceded by a subtle and often lengthy period of symptoms and functional impairment even prior to the first psychotic episode. This was described retrospectively in many studies (Yung and McGorry 1996). The field was transformed in the early 1990s when prospective operational criteria were formulated which were predictive of a high rate of conversion to first episode psychosis from this subthreshold psychotic state termed the ‘at risk mental state’ (ARMS)—later the ultra‐high risk (UHR) or clinical high risk (CHR) state. These criteria were first designed and tested in the EPPIC program in Melbourne at the PACE clinic (Yung et al. 1996). Subsequently a wide range of cohort studies and clinical trials in this population of patients were conducted and psychosis research increasingly incorporated a focus on the earliest clinical stages of illness, among other areas of investigation. These clinical trials have enabled the assembly of a substantial data set concerning the risks and benefits of intervening at this stage of illness, prior to the first psychotic episode. Despite the paradigm shift that this research reflects, recent critiques have questioned the ‘risk and transition’ paradigm (van Os and Guloksuz 2017) from the standpoint of the continuum model; the unnecessary stigmatizing of false positive cases; the choice between universal and indicated prevention from the epidemiologists; and the controversies on the effects of preventive interventions and how to interpret the results of pairwise and network meta‐analyses. This has introduced uncertainty for researchers and clinicians about how best to approach treatment for this clinical population. This paper addresses these uncertainties and challenges. We will discuss how the classification model of distinct psychiatric disorders is challenged by the continuum model in which everything is a variation of normality and the emerging dynamic systems approach to psychopathology with tipping points and transitions between stages. Risk profiles have proven to be helpful in medicine and can also be helpful in psychiatry, but they are considered by some to be unnecessary and stigmatizing. The prevention paradox says that only a minority of cases come from the high‐risk group and that indicated prevention in high‐risk groups do not pay off and furthermore are potentially stigmatizing. We will discuss the use of general prevention and indicated prevention and their costs and effort. Different ways of meta‐analysing have been used with mixed results. NMAs are more powerful than pairwise meta‐analyses (PMA), but the low number of direct comparison and concerns on transitivity may explain the lack of replicating findings from PMAs. The debate on the interpretation of meta‐analytic findings is complex and technical and will be addressed in detail.

## 2 Staging of Psychosis in the Continuum Model Versus Dynamic Systems Model

In the DSM5, over 200 symptoms are part of 265 diagnostic classifications, resulting in a high rate of symptomatic overlap in classification and apparent comorbidity and very little validity for separate disorders. The continuum model is based on the fact that symptoms are also present in the general population: all people have symptoms to a certain extent. A problem with the continuum model is that it has no clear‐cut distinction between ‘normal’ experiences in the population and ‘abnormal’ states of psychopathology. All is a variation of normality. An alternative is the dynamic systems model, which seems more adequate than the distinct disorder model and the continuum model. The dynamic systems model is widely applicable such as to climate, flora and fauna, as well as to networks of psychiatric symptoms (Borsboom et al. 2016; Scheffer et al. 2001). A dynamic system tends to return to equilibrium after disturbance (its ‘basin of attraction’) and tends to slow down in its return to such a stable state after continuous or major disturbance, indicating that it may be closing to a ‘tipping point’. At a tipping point, a dynamic system can rapidly move into a different state of equilibrium (an alternative basin of attraction). These are called catastrophic shifts. The way back is possible, but not easy (Scheffer et al. 2001). The critique of the ‘risk and transition’ paradigm that just variation exists and no discrete stages (van Os and Guloksuz 2017) is less relevant when we consider the behaviour and interaction of symptoms in a dynamic systems model. Just as the stages are conceptualised in oncology, there is also a clear transition from subclinical ARMS to a first episode of psychosis. As the ARMS patient has multiple explanations for the odd experiences (Am I overfatigued? Am I going mad? Did I use too many drugs? Did I sleep too little?), during transition to psychosis the conviction will be a delusional belief (It is the devil speaking to me and I must obey!) and doubt will make place for certainty. The probability of symptom remission diminishes with progression through the stages. Psychotic‐like experiences in the general population (stage 1a) have symptomatic remission at 24 months in 84% of the cases (Hanssen et al. 2005). ARMS in a help‐seeking population in mental health services (stage 1b) has full remission after two years in 46% of the cases (Simon et al. 2013). First episode of psychosis (stage 2) has only one subsequent episode of psychosis within 24 months in 22% of the cases (Fusar‐Poli et al. 2016).

Stages in cancer can be precisely defined and measured; although these stages are arbitrary, they have implications for prognosis and treatment. The transition from prediabetes to diabetes and from prehypertension to hypertension are defined by biomarkers but are also arbitrary; despite that they are arbitrary, they have proven to be helpful in clinical practice for providing preventive intervention. Analysis of symptom‐networks and the dynamic systems model are increasingly applied in psychiatry (Scheffer et al. 2024) and include the continuity of symptoms in the general population and transitions in stages and classification, although the syndromes overlap and have fuzzy characteristics. We consider incompatibility between classification and continuum models as resolved by adapting the dynamic systems model of psychiatric disorder evolution.

Another kind of staging is the sequential development of psychopathology. Most individuals who meet diagnostic criteria for one disorder meet diagnostic criteria for a second disorder; most who meet criteria for two meet criteria for a third; and so forth. Caspi and Moffit stated that ‘…many young children exhibit diffuse emotional and behavioral problems, fewer go on to manifest a brief episode of an individual disorder, still fewer progress to develop a persistent internalizing or externalizing syndrome, and only a very few individuals progress to the extreme elevation of *p*, ultimately emerging with a psychotic condition, most likely during late adolescence or early adulthood’. Empirical evidence has now accrued to suggest that a single dimension is able to measure a person's liability for mental disorder, comorbidity among disorders, persistence of disorders over time and severity of symptoms. This single dimension of general psychopathology has been termed the general psychopathology factor or ‘*p*’ (Caspi and Moffitt 2018). Studies showed that the higher a person scores on p, the worse that person fares on measures of family history of psychiatric illness, brain function, childhood developmental history and adult life impairment (Caspi and Moffitt 2018; Cervin et al. 2021).

To conclude we suggest that the dynamic systems model integrates the classification model with the continuum model as it recognises disorders as overlapping and fuzzy sets of interacting symptoms and justifies stages that are clearly demarcated from each other.

## 3 Stigma

The second controversy is on stigma. A valid objection to preventive medicine is potential stigmatisation. Both ARMS‐status and symptom experiences may catalyse stigmatisation by peers, family, school and job associates, health providers and one's self (DeLuca et al. 2021). However, we know of no studies where stigma associated with ARMS status is greater than stigma associated with any other disorder and stigma already occurs before help‐seeking (Yung et al. 2021). If patients fulfil the criteria of ARMS and are told so, this can also be beneficial because of the validation of odd experiences, relief of not being psychotic and being connected to specialised services (Uttinger et al. 2018). Stigmatisation can be diminished by normalising odd experiences and stressing the opportunities to prevent adverse outcomes and retain functioning in society (Sol‐Nottes et al. 2024). Location of services for at risk individuals within low stigma environments, such as Youth Mental Health services, further reduces stigma (Yung et al. 2021). Also, CBT for ARMS should encourage patients to continue school and workplace as dropout will be an extra barrier in recovery. If necessary, outreach to school and workplace may be provided to preserve social functioning and integration in society (van der Gaag, Nieman, and van den Berg 2013).

Another way of limiting stigma is to integrate patient and professional perspectives on terminology. In a recent study, the preferred terms were ‘pre‐diagnostic stage’, ‘potential of developing mental illness’, and ARMS. The least favoured terms were UHR and Attenuated Psychosis Syndrome, which were thought to be associated with the most stigma by all participants. Most participants agreed that disclosure about diagnosis should be delivered early by the key clinician (Polari et al. 2021).

Ultimately, stigma is not only a problem of the mental health system, but the attitude of the general public and it is not a potent enough reason to withhold care. Destigmatisation must also be accomplished at the community level by media, school education, politics and legislation.

## 4 Universal Versus Indicated Prevention

A third controversy is on the prevention paradox. This refers to the scenario of most cases of a disorder arising in a low‐risk group, with a minority in a high‐risk group and therefore a successful public health intervention would result in greater benefit than indicated prevention (Guloksuz et al. 2020). However, we do not stop treating high blood pressure in medical practice due to most heart attacks occurring in people without hypertension. In our view, the paradox is not in fact a paradox and leaves the option of combining indicated prevention and intervention at the population level (John 2011). Benefit–cost ratios need to be developed to inform policymakers. Furthermore, public health campaigns are extremely expensive and are not always cost‐effective, while indicated prevention in ARMS patients is cost‐saving at 18‐ and 48‐month follow‐up (Ising et al. 2015, 2017).

In fact, the vast majority of individuals (78.3%) experience a prodrome prior to a first episode of psychosis (Benrimoh et al. 2024). However, the problem is that they are not being identified. The Oasis project in London only saw 4.1% of FEP patients before they developed psychosis (Ajnakina et al. 2017), which is a small proportion. In Melbourne, 21.3% of FEP patients were referred by youth services; 13.7% of these FEP cases had attended the ARMS clinic (Burke et al. 1999). Given the high prevalence of transition from prodrome to psychosis, it seems sensible to combine screening‐based indicated prevention in mental health with public education and extra training for professionals to build awareness and timely referral for intervention (Dodge 2020). Also, the prevention of childhood abuse, partner violence, substance abuse and homelessness and securing economic participation and fostering social support are interventions on a population level that can prevent psychosis (Oswald et al. 2024).

Although ARMS status has a strong valence for the development of a first psychotic episode (Webb et al. 2015), ARMS status together with psychotic‐like experiences and isolated psychotic symptoms is also a severity index for comorbid disorders. For example, depression with ARMS responds less successfully to depression therapy than depression without ARMS (Smith et al. 2009; Wigman, van Os, et al. 2014) and ARMS subjects are also at risk for poor outcome (Wigman, Devlin, et al. 2014), suicidality (Saha et al. 2011) and long‐term mental health trajectories (Trotta et al. 2020).

## 5 Overview of Meta‐Analyses of Treatment Effects

The fourth controversy is on the interpretation of meta‐analytical evidence on prevention of psychosis. The meta‐analyses published until now that have included transition to psychosis as an outcome are shown in Table 1. We discuss the characteristics of the meta‐analyses concerning inclusion and exclusion of trials and the effects on the results.

**TABLE 1:** Results of PMA and NMA on the efficacy of CBT for ARMS in reducing transition to psychosis.

Meta‐analysis	Type of meta‐analysis	6‐month	12‐month	18‐month	24–48‐month
Stafford et al. (2013)	PMA (completers only)	*k* = 4; *n* = 598; RR = 0.62 (0.29–1.31), *p* = 0.06, *I* ^2^ = 17	*k* = 5; *n* = 645; RR = 0.54 (0.34–0.86), *p* = 0.009, *I* ^2^ = 0	*k* = 4; *n* = 570; RR = 0.63 (0.40–0.99), *I* ^2^ = 0	
van der Gaag, Smit, et al. (2013)	PMA (CBTarms only; non‐reported data)		*k* = 5; *n* = 672; RR = 0.52 (0.32–0.82), *p* = 0.006, *I* ^2^ = 0		*k* = 3; *n* = 549; RR = 0.64, (0.41–0.99) *p* = 0.046, *I* ^2^ = 0
Hutton and Taylor (2014)	PMA (Including IPI study)	*k* = 6; *n* = 800; RR = 0.52 (0.27–1.02) *p* = 0.06, *I* ^2^ = 13	*k* = 6; *n* = 800; RR = 0.45 (0.28–0.73) *p* = 0.001, *I* ^2^ = 0	*k* = 6; *n* = 452; RR = 0.41 (0.23–0.72) *p* = 0.002, *I* ^2^ = 0	
Schmidt et al. (2015)	PMA (Including IPI, FI, OLA RIS and Om3)	*k* = 7; *n* = 916; RR = 0.36 (0.21–0.60), *p* = 0.0001, *I* ^2^ = 0, Including 1 IPI and 1 FI	*k* = 10; *n* = 1071; RR = 0.44 (0.31–0.61), *p* = 0.00001, *I* ^2^ = 0, Including 1 IPI, FI, 1 OLA, 1 RIS, 1 Om3	*k* = 5; *n* = 595; RR = 0.58 (0.40–0.85) *p* = 0.005, *I* ^2^ = 0, Including OLA	*k* = 4; *n* = 668; RR = 0.41 (0.25–0.69) *p* = 0.0007, *I* ^2^ = 34
Davies et al. (2018)	NMA (CBTarms divided into three nodes)	*k* = 7; no superiority	*k* = 7; no superiority		
Bosnjak Kuharic et al. (2019)	PMA		*k* = 5; *n* = 728; RR = 0.47 (0.29–0.76), *p* = 0.001, *I* ^2^ = 0, Including IPI	*k* = 2; *n* = 253; RR = 0.45 (0.23–0.89) *p* = 0.02, *I* ^2^ = 0	*k* = 1; *n* = 128; RR = 0.32 (0.11–0.92), Including IPI
Devoe et al. (2020)	PMA	*k* = 6; *n* = 729; RR = 0.66 (0.33–1.34), *p* = 0.25, *I* ^2^ = 19	*k* = 6; *n* = 729; RR = 0.57 (0.35–0.93) *p* = 0.02, *I* ^2^ = 7	*k* = 3; *n* = 540; RR = 0.54 (0.32–0.92) *p* = 0.02, *I* ^2^ = 7	*k* = 3; *n* = 549; RR = 0.69 (0.35–1.70), *p* = 0.11, *I* ^2^ = 0
	NMA	*k* = 6; no superiority	*k* = 6; no superiority		
Mei et al. (2021)	PMA (non‐reported data at 18 and 24–48 months)	*k* = 7; *n* = 787; RR = 0.57 (0.29–1.13), *p* = 0.106; *I* ^2^ = 23	*k* = 7; *n* = 787; RR = 0.53 (0.33–0.82), *p* = 0.005; *I* ^2^ = 6	*k* = 3; *n* = 540; RR = 0.53 (0.31–91), *p* = 0.022; *I* ^2^ = 0	*k* = 3; *n* = 549; RR = 0.64 (0.41–0.99), *p* = 0.046; *I* ^2^ = 0
	(reported data)			*k* = 6; RR = 0.60 (0.42–0.84), *p* = 0.004; *I* ^2^ = 0	
Zheng et al. (2022)	PMA	0–6 months *k* = 7; *n* = 839; RR = 0.52 (0.26–1.01) *p* = 0.06, *I* ^2^ = 31	6–12 months *k* = 9; *n* = 1025; RR = 0.44 (0.30–0.64) *p* < 0.0001, *I* ^2^‐0	12–24 months *k* = 5; *n* = 778; RR = 0.46 (0.30–0.69) *p* = 0.0002, *I* ^2^ = 0	24plus months *k* = 3; *n* = 319; RR = 0.58 (0.35–0.95) *p* = 0.03; *I* ^2^ = 0
Minichino et al. (2025)	PMA, including studies with other primary outcomes	*k* = 10; OR = 0.84 (0.52–1.35), *p* = 0.47, *I* ^2^ = 0	*k* = 9; OR = 0.64, (0.39–1.06), *p* = 0.08, *I* ^2^ = 0.21	*k* = 3, OR = 0.49, (0.27–0.90), *p* = 0.02, *I* ^2^ = 0	*k* = 2; OR = 0.58 (0.31–1.07), *p* = 0.08, *I* ^2^ = 0

*Note: k* = number of studies; *n* = number of subjects; *I* ^2^ = heterogeneity.

Abbreviations: FI, family intervention; IPI, Integrated Psychological Intervention; OLA, olanzapine; Om3, omega‐3 fatty acid; RIS, risperidone; RR, risk ratio (and 95% confidence interval).

An early meta‐analysis was by Stafford and colleagues. The results were a significantly reduced risk for conversion to psychosis for CBT at 12 and 18‐months (Stafford et al. 2013).

Another meta‐analysis from 2013 combined all interventions (3 on antipsychotic medication, 1 trial on omega‐3 fatty acid, 2 trials on integrated psychological interventions and the same 5 CBT for ARMS studies as included in Stafford et al. (2013)) in one meta‐analysis and found that conversion rates are about halved at 12 months and were reduced by a third at 2‐ to 4‐year follow‐up (van der Gaag, Nieman, and van den Berg 2013) compared to any control condition. There was some indication of publication bias with Duvall and Tweedie's trim and fill procedure, but the results remained statistically significant.

One year later (2014) a new meta‐analysis appeared (Hutton and Taylor 2014). This meta‐analysis no longer included the OPUS trial as that sample consisted of schizotypal patients, some of whom were already on antipsychotic medication at baseline (Nordentoft et al. 2006). The analysis had six trials on CBT for ARMS, including the EIPS trial on integrated psychological therapy (IPI) that combined individual CBT for ARMS with some group skills training, cognitive remediation and multifamily education (Bechdolf et al. 2012). Six studies contributed to the 6‐month outcome; the RR = 0.52 was not significant in the random effects meta‐analysis. The findings at 12‐months and at 18–24 months were both statistically significant.

The European Psychiatric Association (EPA) provides evidence‐based recommendations in guidelines. They published a guidance paper on early intervention in CHR based on a meta‐analysis of 15 studies (Schmidt et al. 2015). The EPA also considers that in adult CHR patients a staged intervention model should be applied with the least restrictive service approach, that is, CBT for ARMS, being offered as a first choice (Schmidt et al. 2015).

The first NMA published on the prevention of psychosis appeared in 2018 (Davies et al. 2018). The advantage of NMA is that interventions can be estimated and compared that have never been tested in head‐to‐head studies. Differences in effect‐sizes are calculated by indirect evidence, for example, if two medications have both been compared to placebo, the difference between the two interventions can be estimated by their relative effects compared to placebo. NMA can in this way gain statistical power. NMA can also rank efficacy and tolerability of different interventions. The results were that no intervention was significantly superior to other interventions in preventing psychosis at 6‐ and 12‐months (Davies et al. 2018). This does not mean that the interventions are not effective. The NMA only included 16 studies and only 20 comparisons between 11 treatment nodes. Almost all comparisons had only one or two comparisons and only four indirect comparisons between treatments were available. Most meta‐analysts would say that in this case a NMA should not be conducted because of a lack of primary studies. In the discussion the authors mention that large confidence intervals are observed and that sizeable effects may still have been missed. The authors also state that many nodes were not well connected, with the corollary of limited ability to check for inconsistency, more imprecise estimates and wide 95% confidence intervals. Despite these limitations, the ranking mirrors the findings from PMA, with IPI, family intervention and CBT for ARMS in general to be more effective than pharmacological interventions.

The Cochrane review from 2019 selected only five somewhat older CBT for ARMS studies with a minimum of 200 participants that contrasted with supportive therapy and was questioned for the assessment of the quality of the studies and downplaying good results for CBT for ARMS in the final recommendation (McGorry and Nelson 2020). Although their general conclusions were negative about the possibilities of preventing psychosis, they did report PMA statistically significant effects for CBT for ARMS at 12, 18 and 24 months (see page 182) (Bosnjak Kuharic et al. 2019).

Another meta‐analysis reported PMA and NMA results (Devoe et al. 2020). The PMA reflected the effect sizes on 12‐ and 18‐months for CBT for ARMS that have been reported before. The 24–48 months results were not statistically significant. The NMA results showed no statistically significant superiority of any treatment over any other treatment. Again IPI, family intervention and CBT for ARMS were ranked somewhat higher. The authors suggested that if a patient presents with being at risk for psychosis, clinicians should consider offering CBT for ARMS to help reduce the risk of a first episode.

It is intriguing that the two NMAs did not confirm the findings of the numerous PMAs. NMA can only result in valid data when the requirement of transitivity is met. An important criterion for transitivity is that participants in each included trial should in theory have been consenting to participate in every other included trial. This is unlikely to be the case in the current scenario, because most individuals with an ARMS prefer certain treatments. Most patients (57.2%) would have preferred to be randomised to CBT, 16.7% preferred Case Management + Aripiprazole, 0.7% chose Case Management + Placebo and 25.4% stated that they had no preference regarding allocation (Bechdolf et al. 2023). Furthermore, most interventions were single trials so that the indirect evidence could only be partially checked for consistency with direct evidence. Transitivity in this context is therefore at least uncertain but probably the assumption of transitivity is not met. A violated transitivity assumption results in invalid indirect effects and erroneous adjustments to direct effects. The NMA approach remains premature at this stage for trials in the ARMS field. That means that when transitivity is as uncertain as it is here, the best solution is to stick with the direct, causal evidence from PMAs.

A PMA on all interventions aimed at reducing conversion to psychosis also reported CBT for ARMS trials (Mei et al. 2021). Pooling all seven pharmacological interventions (olanzapine, ziprasidone, D‐serine, glycine and omega‐3 fatty acids) showed no significant reduction of conversion to psychosis at any time‐point. Pooling all 11 psychological interventions did show statistically significant reductions in the conversion rates at all time points. CBT for ARMS was not significant at 6‐months, but did show significant effects at end‐of‐treatment, 12‐months and 18‐plus months. For the sake of comparability, we present in Table 1 the results at 18 months and 24–48 months, which are also both statistically significant.

Several issues were discussed about this meta‐analysis (Fusar‐Poli, Radua, and Jauhar 2021). The first objection was that Mei et al. (2021) used other conversion data in the Morrison study (Morrison et al. 2007, 2004) compared to other meta‐analyses. The Morrison et al. study reported three outcomes for conversion: PANSS conversion (2 versus 5 cases), prescription of antipsychotic medication (2 versus 7 cases) and DSM‐IV diagnosis of psychotic disorder (2 versus 6 cases). After consulting the authors of the primary study, the meta‐analysis used the DSM‐diagnosis, which is not the most favourable, but also not the least favourable result for CBT for ARMS. The second objection was that the unpublished PREVENT study was not included (Bechdolf et al. 2011). The study's exclusion was at the request of the authors of the PREVENT study as preliminary publication may have obstructed the publication process of the original data. The third objection was the inclusion of a recent study by Pozza and Dèttore (2020), which was suggested to be excluded as it was not registered and has a high risk of bias. Mei and colleagues contacted Pozza and the reported conversions were verified to be conversions to full‐blown psychosis and lasting more than a week. The meta‐analysis used the raw conversion data of all used studies.

Further subgroup analyses as suggested in the recent letter to the editor (Fusar‐Poli et al. 2022) have been conducted. Adding the PREVENT study, omitting the Pozza and Dèttore study and choosing the ‘worst’ outcome for Morrison et al. (2004) did not change the 12‐month results.

The meta‐analysis by Zheng and colleagues showed comparable results (Zheng et al. 2022), but was criticised for being overoptimistic and not robust (Fusar‐Poli et al. 2022). It included trials that had not been pre‐registered and data may have been extracted incorrectly.

The most recent meta‐analysis is by Minichino et al. (2025), which only found effects at 18 months. They excluded two effective trials for not being pre‐registered, but included two trials that were also not preregistered (EDIE‐UK and ADAPT). The PRISMA guideline for conducting a meta‐analysis does not demand that studies are preregistered. Sensitivity analyses can effectively deal with different levels of risk of bias.

They also used a broad CBT selection principle, instead of CBT targeted at the prevention of transition. A trial was included that used modular CBT in a case management framework (CBCM) that was adapted to improve functional recovery (McGorry et al. 2023) and another study that investigated group cognitive‐behavioral social skills training (CBSST) to improve social and role functioning (Addington et al. 2023) and did not aim to reduce transition as a primary outcome. This meta‐analysis also used Odds Ratios that diverge from Risk Ratios as incidence increases. Odds Ratio is a good choice in case–control studies, while Risk Ratio is the preferred choice in randomised controlled trials. The authors did not give a reason for their deviation from the conventional use of Risk Ratio.

Over time we see that the meta‐analyses include more studies. In the beginning, all kinds of interventions were pooled together, but with more trials, interventions were separately evaluated. In PMAs, the effects of CBT for ARMS stay about the same over time. The effects decline a little bit from RR = 0.50 to RR = 0.40, but because of more statistical power from more studies, the level of statistical significance gets more convincing. Because many interventions have only one published trial, it seems preliminary to conduct NMA as transitivity cannot be tested properly and confidence intervals are huge. More trials are needed for more robust findings.

Very often a meta‐analysis is updated after several years, while many trials may have been published. The risk is that the recommendation of the meta‐analysis is outdated. A living cumulative meta‐analysis updates after every study. A cumulative meta‐analysis can also set the point where the pooled findings are robust and no further trials are needed. In CBT for psychosis (not an ARMS population), it was shown that the effect sizes stabilised in delusions in 2015 and in hallucinations in 2016. The effect sizes did not change anymore (Turner et al. 2020). In CBT for ARMS, an a priori success criterion was set to a risk ratio reduction of 0.50. No clear reasons were given for such a high criterion (Fusar‐Poli et al. 2019). However, our view is that this threshold is set unrealistically high with a risk ratio reduction of 0.50 or less for CBT for ARMS. Applying these threshold criteria to other domains, such as prevention of cardiac disease, would mean that most state‐of‐the‐art treatments would fail. This would certainly be the case for the use of statins (Chou et al. 2016) as well as atrial fibrillation catheter ablation (Saglietto et al. 2020). However, these treatments are part of standard, evidence‐based clinical practice all over the world as benefits outweigh the potential harms.

## 6 Concluding Remarks

Four controversies have been discussed in this paper: (1) models of conceptualising psychopathology and stages; (2) stigma and how to deal with this; (3) the prevention paradox and issues of universal and/or indicated prevention; and (4) the interpretation of the experimental data in favour of prevention of psychosis by CBT.

### 6.1 The Staging Model

The staging model of psychosis is supported by the emerging dynamic systems model of interacting psychopathology. This model describes how sudden shifts in psychopathology can occur and how it is used, for instance, in oncology and climate change and that it can also be applied to stages of psychosis ranging from perceptual anomalies in the general population to psychotic episodes and chronic psychosis in progressed stages.

### 6.2 Stigma

Stigma is an issue, as is in many diseases and disorders. Most patients in specialised services appreciate the help and do not feel stigmatised by professionals and are helped to overcome self‐stigma. Normalising anomalous experiences by highlighting that they are highly prevalent in the general population helps to destigmatise and at the same time help people with at risk status to remit. Also, specialised services not associated with schizophrenia health care are less stigmatising.

### 6.3 The Prevention Paradox

The risk of developing a first psychotic episode increases manifold from 0.02% per year in the general population to 15% in the first year in the ARMS group to 28% after four and more years (Salazar de Pablo et al. 2021). This high‐risk group can be successfully and cost‐effectively treated to a clinically meaningful extent with CBT for ARMS. Other interventions are promising.

A large part of the population is not liable to develop psychosis at all and forms the no‐risk group (van Os and Linscott 2012; Linscott and van Os 2010). The low‐risk group (stage 1a with psychotic‐like experiences, normal functioning and no help‐seeking behaviour) with a transition rate to psychosis of 0.6% is the group in the community that may be addressed by population‐based preventive interventions, but no data are available on effective interventions at this moment. At the same time, those who advocate a public health approach now also agree with the idea of a broad approach with help‐seeking persons for non‐psychotic disorders and subclinical psychosis to improve outcomes in a broad sense in a non‐stigmatizing youth mental health setting (Guloksuz et al. 2020; Hasmi et al. 2021).

### 6.4 The Interpretation of the Scientific Evidence

Concerning the effects of CBT for ARMS it seems that the two trials by van der Gaag, Nieman, and van den Berg (2013) and Pozza and Dèttore (2020) showed the largest effects. These trials used the same CBT for ARMS protocol that specifically targeted the prevention of delusion formation based on perceptual aberrations, incidental hallucinations and suspiciousness.

At the same time, CBT combining individual CBT with group skills training, cognitive remediation and multifamily psychoeducation had very good results (Bechdolf et al. 2012). We need more trials that specifically target prevention of transition and trials that integrate effective therapies for psychosis.

Another issue is how to set standards for effective interventions to reduce the conversion rate to psychosis. In the case of the effectiveness of CBT for ARMS in reducing conversion rates, the effects have stayed stable over time in PMAs. At the moment, there is evidence in PMAs for large reductions of conversion to psychosis of about 40% at 12‐months with limited evidence of publication bias and low evidence of heterogeneity and a 36% reduction of conversions at 24 to 48‐months follow‐up with also low evidence of publication bias and low evidence of heterogeneity (see Table 1). The evidence is robust after correction for publication bias and removing outliers in PMAs. Those who developed psychosis in the CBT for ARMS condition were in general adherent to continued treatment with antipsychotic medication. CBT for ARMS socialised them in talking about odd experiences and attenuated psychotic symptoms and knowing that there is a small risk of developing psychosis. This is in contrast with people with a first episode of psychosis who are in general denying a psychotic condition and are reluctant to take medications as they consider their condition as overstressed and non‐psychotic. Even if prevention intervention failed in ARMS patients, they have a very short duration of untreated psychosis (DUP) and a better prognosis, while an extended DUP is associated with long‐term symptoms.

NMA can be important for clinical decision making as it ranks the relative superiority and tolerance of interventions. However, in NMAs with many interventions that have only been tested in a single small trial with limited testing of consistency between direct and indirect evidence, the differences between interventions are drowned in huge confidence intervals. NMA has been conducted for quite some years and has clear rules, but the NMAs that we reviewed in this paper have not been conducted in accordance with these rules well enough.

The research agenda is that more trials are needed. CBT for ARMS is recommended in several guidelines and antipsychotic medication is not recommended at this moment. While the evidence for CBT for ARMS is growing, pharmacological interventions such as olanzapine, ziprasidone, D‐serine, cannabidiol and glycine each have only one trial published. To support the feasibility of NMA, many more trials with head‐to‐head comparisons are required.

Possibly adding family intervention and skills training, including (online) social interventions, may further improve functioning (Alvarez‐Jimenez et al. 2018) and omega‐3 fatty acids may be helpful as a personalised additional treatment for the subgroup with low levels of this neuroprotective agent (Amminger et al. 2020). There are also trials of other interventions running at this moment and all are directed at improving quality of life in help‐seeking adolescents and young adults to preserve functioning and hope for the future.

Conversion to psychosis remains a justified target (Fusar‐Poli et al. 2020), but at the same time early intervention also needs to address other goals and areas of clinical need such as PTSD, anxiety, depression, social functioning and participation in society, as existing UHR interventions have proven to be less effective for these domains (Devoe et al. 2019; Mei et al. 2021). Also, the highly effective integrated treatment packages need to be replicated. If interventions do not prove effective or are no longer effective, the field has to move on, adopt protocols to enhance efficacy and develop new interventions to help young people in despair to regain hope, functioning and a promising future without poor long‐term mental health trajectories (McGorry and Killackey 2002; Fusar‐Poli, Radua, and Jauhar 2021).

While CBT for ARMS and other psychosocial treatments has beneficial effects for high‐risk patients, they are only partially effective and not appropriate for all cases. Hence, we share the hope that the future will bring a greater array of effective interventions in the prevention of psychosis, as well as a more personalised approach. An improved understanding of the heterogeneity of ARMS individuals, biomarkers that identify increased risk for various outcomes (transition from stage 1a to 1b to conversion, prolonged poor functioning, negative symptoms for example) and strategies to enrich risk and stratification of risk groups are all methods that will improve our ability to discover new effective treatments and enable personalised treatment (Wannan et al. 2024).

## Funding

The authors have nothing to report.

## Data Availability

Data sharing not applicable to this article as no datasets were generated or analysed during the current study.
